# Instituting systems-based practice and practice-based learning and improvement: a curriculum of inquiry

**DOI:** 10.3402/meo.v18i0.21612

**Published:** 2013-09-16

**Authors:** Andrew P. Wilper, Curtis Scott Smith, William Weppner

**Affiliations:** 1 Division of General Internal Medicine, Department of Medicine, University of Washington, Seattle, WA, USA; 2 Boise VA Medical Center, Boise, ID, USA

**Keywords:** graduate medical education, competencies, longitudinal curriculum

## Abstract

**Background:**

The Accreditation Council for Graduate Medical Education (ACGME) requires that training programs integrate system-based practice (SBP) and practice-based learning and improvement (PBLI) into internal medicine residency curricula.

**Context and setting:**

We instituted a seminar series and year-long-mentored curriculum designed to engage internal medicine residents in these competencies.

**Methods:**

Residents participate in a seminar series that includes assigned reading and structured discussion with faculty who assist in the development of quality improvement or research projects. Residents pursue projects over the remainder of the year. Monthly works in progress meetings, protected time for inquiry, and continued faculty mentorship guide the residents in their project development. Trainees present their work at hospital-wide grand rounds at the end of the academic year. We performed a survey of residents to assess their self-reported knowledge, attitudes and skills in SBP and PBLI. In addition, blinded faculty scored projects for appropriateness, impact, and feasibility.

**Outcomes:**

We measured resident self-reported knowledge, attitudes, and skills at the end of the academic year. We found evidence that participants improved their understanding of the context in which they were practicing, and that their ability to engage in quality improvement projects increased. Blinded faculty reviewers favorably ranked the projects’ feasibility, impact, and appropriateness. The ‘Curriculum of Inquiry’ generated 11 quality improvement and research projects during the study period. Barriers to the ongoing work include a limited supply of mentors and delays due to Institutional Review Board approval. Hospital leadership recognizes the importance of the curriculum, and our accreditation manager now cites our ongoing work.

**Conclusions:**

A structured residency-based curriculum facilitates resident demonstration of SBP and practice-based learning and improvement. Residents gain knowledge and skills though this enterprise and hospitals gain access to trainees who help to solve ongoing problems and meet accreditation requirements.

## Introduction

The Accreditation Council for Graduate Medical Education (ACGME) requires that training programs integrate system-based practice (SBP) and practice-based learning and improvement (PBLI) into residency curricula. SBP requires a demonstrated awareness of and responsiveness to the larger context and system of healthcare, as well as the ability to call effectively on other resources in the system to provide optimal healthcare. Residents demonstrate PBLI by self-directed learning and continuously improving patient care (1). Both have been described as conceptually difficult for residents (2). To enhance our internal medicine residency program's delivery of these competencies, we instituted a curriculum to demonstrate the need for and value of original investigation and quality improvement. Our aim was to fulfill the ACGME requirement of instituting SBP and PBLI.

## Methods

### Setting/participants

Eight second-year residents and three interns in internal medicine participated in the curriculum during the 2010–2011 academic year at the Boise VA Medical Center. None of these participants had formal training in quality improvement, and all had a background in basic science research.

### Intervention – the curriculum of inquiry

We begin with a series of eight 2-hour interactive seminars occurring in the first quarter of the academic year supported by required readings (Table 1). We arrived at the reading list following a review of the Achieving Competency Today (ACT) curriculum bibliography, provided by Dr. Antionette Peters (3). In addition, the course director performed a PubMed search and obtained recommended articles from course faculty capturing the major themes of the seminars. During the introductory session, we review trainees’ prior research experience. In this session, we aim to develop interest in a question or challenge that can be formed into a defined research or quality improvement project. We focus on the patient–provider relationship and spheres of influence that impact that dyad. Using a series of concentric circles, we build a model for these relationships. We note how these domains correlate to the ACGME competencies, and how research and quality improvement are fundamental in the process of improving the quality of patient care and patient care systems at all levels.

**Table 1:** Curriculum of inquiry seminar series outline

Seminar title	Content	Pre-reading
Course introduction	Discuss problems with patient care that the residents have encountered in their training. Focus this discussion on elements of the healthcare system that failed. Introduce ecologic model concept and discuss with the different forces at play influencing how they can care for their patients. Distribute inquiry benchmarks and description of inquiry forms.	Berwick D. A. Primer on leading the improvement of systems. BMJ: 1996;312;619–22 Cutler D: Your Money or Your Life: Strong Medicine For America's Health Care System. Oxford University Press; 2004. Donabedian, A. The Quality of Care. How Can it Be Assessed. JAMA: 1988;260;1743–48
System financial incentives	Discuss system-wide financial incentives. Include payors, providers, hospitals	Bodenheimer T. High and Rising Health Care Costs. Part 1. Ann Intern Med. 2005;142:847–854. Bodenheimer T. High and Rising Health Care Costs. Part 2: Technologic Innovation. Ann Intern Med. 2005;142:932–937. Bodenheimer T. High and Rising Health Care Costs. Part 3: The Role of Health Care Providers. Ann Intern Med. 2005;142:996–1002. Bodenheimer T. High and Rising Health Care Costs. Part 4: Can Costs Be Controlled While Preserving Quality. Ann Intern Med. 2005;143:26–31. Gawande A. The Cost Conundrum. What a Texas town can teach us about health care. June 1, 2009. New Yorker.
Project development	Review trainee's proposals. Begin outreach to parties most interested in or affected by any quality improvement proposals	Ogle B. Asking the Research Question. Fam Prac Res Jour. 1984:4:8–14. Greco: Changing Physicians’ Practices. New Eng J Med. 1993;329:1271–74 Berwick, Disseminating Innovations in Health Care. JAMA. 2003:289:1969–75.
Library search	Session with a medical librarian who has searched key phrases submitted by residents related to project proposals.	Ebbert J. Searching the Medical Literature Using PubMed: A Tutorial. Mayo Clin Proc. 2003;78:87–91 Sood A. Using Advanced Search Tools on PubMed for Citation Retrieval Mayo Clin Proc. 2004;79:1295–1300 Dupras D. Clinicians’ Guide to New Tools and Features of PubMed. Mayo Clin Proc. 2007;82:480–484
Evidence-based medicine	A practical review of the use of the statistics and the medical literature.	C. Scott Smith, MD White paper (unpublished)
Case-specific methodology	Project development	None
Guest speaker	Content expert invited to address seminar	
Project proposals	Final submission of written proposals that will be pursued during the remainder of the academic year	

Next, we focus on system-level financial incentives that influence patient care. During the following seminar, we hone the residents’ initial research or quality improvement question. During this discussion, three faculties help shape project ideas into feasible projects. We consider those projects whose ultimate aim is peer-reviewed publication as research and label those aimed at improving clinical systems within our hospital to be quality improvement. We request that trainees perform a literature review to discover what is already known about their topic of interest. During the following seminar, a medical librarian demonstrates search techniques and results from trainees’ queries. We compare results from the two search approaches to improve search skills. We dedicate the subsequent seminar to demonstrating a practical approach to interpreting statistics and the medical literature. This session focuses on several clinical questions, demonstrated by recent published studies. The trainees’ current practice and the conclusions of the studies are compared to demonstrate this approach. Next, we focus on case-specific methodology. During this seminar, we distribute structured project development templates along with local institutional review board (IRB) documentation for project submission. In addition, we ask that trainees complete a project proposal abstract. We invite project stakeholders (e.g., data manager and statistician) to attend to review feasibility of study design. Trainees select faculty mentors who guide and review project progress over the course of the year. During the penultimate seminar, we invite a resident project-specific content expert to address the group to enhance knowledgebase and project development. Finally, the residents present their project proposals for the remainder of the year.

Following the seminars, trainees schedule a 4-week scholarship block during which they continue work on their project. The course director reviews and approves the goals and objectives for the rotation. Program-wide ‘works in progress’ meetings that include all program residents and faculty mentors are held monthly for trainees to present their work. At the end of the academic year, trainees present the results to-date of their research or quality improvement project to the faculty and staff at the Boise VAMC as a grand rounds presentation. Trainees are encouraged to submit their work at regional and national meetings for presentation.

### Analysis

Following the year-long 2010–2011 curriculum, we asked residents to rate their skills and attitudes in a pre-post questionnaire format, using a 1–5 Likert ranking and paired *t*-tests for statistical significance. Trainees estimate skills and attitudes prior to the curriculum and at the time of the survey. We hypothesized that the curriculum would lead to improvements in these self-assessments. For this questionnaire, we adapted questions from a survey by Yedidia and Peters (3, 4). Finally, we asked three blinded faculties to review and score each project's summary with regard to feasibility (not to very), impact on system change (little to high), and appropriateness of the intervention (not to very), on a 1–5 Likert scale. The Puget Sound VAMC IRB approved this project. We used SAS 9.1 (Carey, NC) for all statistical analyses. This material is the result of work supported by resources from the Boise VA Medical Center, Boise, ID.

## Results

We developed five research and six quality improvement projects (Table 2). Exemplar work included a quality improvement project entitled ‘Improving Transitions of Care from the Inpatient to Outpatient Setting at the Boise VA’, in which a resident identified that only in one-third of cases is a patient's primary care provider (PCP) alerted to a patient's discharge summary following hospitalization. This project resulted in a lengthy review process at the national level, and a service request to modify the Veterans Administration electronic medical record nationwide that would allow PCPs to be added as additional signers to all of their primary care panel's discharge summaries. In another case, IRB approval was granted to a project using the Community Tracking Survey to analyze the relationship between physician work hours and career satisfaction. An abstract from this project was selected for oral presentation at a regional scientific meeting, winning best scientific abstract.

**Table 2:** Changes in self assessed competency following the curriculum of inquiry

Question	Beginning of year, mean, standard deviation (SD) *n*=11	Now, mean, (SD) *n*=11	*p*, paired *t*-test
Locate and critically evaluate research evidence and apply conclusions of the care of an individual patient or group.	3.0 (0.67)	4.0 (0.47)	*p*=0.001
Update the knowledge and skills of colleagues.	2.9 (.57)	4.0 (0.47)	*p*<0.0001
Identify clinical conditions appropriate for quality improvement projects and participate in implementation.	2.8 (0.63)	4.4 (0.70)	*p*=0.0002
Evaluate referrals to specialists for appropriateness and quality, and indicate strategies for improving their effectiveness.	2.6 (0.52)	4.1 (0.57)	*p*<0.0001
Speak publicly about appropriate tradeoffs between costs and quality in the formulation of insurance plans.	2.6 (0.52)	3.0 (1.1)	*p*=0.015
Conduct time and workflow analyses to enhance productivity.	2.5 (0.71)	3.0 (1.05)	*p*=0.01
Predict the impact of different payment arrangements on consumer and provider behaviors within a specific healthcare environment.	2.7 (0.48)	3.0 (0.47)	*p*=0.08
Articulate the strengths and weaknesses of the single-payer Canadian healthcare system and the American healthcare system.	2.5 (0.85)	3.1 (1.1)	*p*=0.02
Explain the reasons for a decision to allocate resources to serve the needs of populations at the potential expense of individual needs.	2.6 (0.52)	3.4 (0.84)	*p*=0.01

Internal medicine resident responses to the following scenario: rate your current competence to do the following: 1–5 Likert scale; 1=not competent; 3=somewhat competent; 5=highly competent.

Residents reported improvement in nearly all of the domains of knowledge and self-rated competency tested. The most dramatic improvements were reported to the following self-rated abilities: ability to identify clinical conditions appropriate for quality improvement projects and participate in implementation: pre=2.8 (SD= 0.63), post=4.4 (SD=0.70), *p*<0.001, and evaluate referrals to specialists for appropriateness and quality, and indicate strategies for improving their effectiveness, pre=2.6 (SD=0.52), post=4.1 (SD=0.57), *p*<0.001 (Table 3). Blinded faculty reviewers favorably scored the projects with regard to feasibility, impact, and appropriateness (Table 2).

**Table 3:** Curriculum of inquiry project and blinded faculty review assessments*

Project category and title	Feasibility of design, mean (SD), *n*=3	Impact on system change, mean (SD), *n*=3	Appropriateness of the evaluation, mean (SD), *n*=3
Quality improvement			
Analysis of the chronic pain pathway	4.7 (0.6)	3 (1)	4.3 (0.6)
Reportable illness reporting at the Boise VA	3 (1)	2.7 (0.6)	2 (1.4)
Relationship between gabapentin use and suicidality	4 (1)	3.7 (1.2)	3.7 (1.5)
Introduction of a novel tool for cardiovascular risk stratification to an ambulatory care clinic	3 (2.8)	3 (0)	4 (1.4)
Improvement of inpatient to outpatient transitions of care at the Boise VA	4.3 (0.6)	4.3 (1.5)	4.3 (1.5)
Predictors of admission to Boise VA Internal Medicine Service for patients not meeting interqual criteria	5 (0)	3.5 (0.7)	4.5 (0.7)
Research			
Internal medicine student rotation grading: does duration of time spent with the attending matter?	4.3 (1.5)	3.7 (1.7)	4 (1)
A sensitivity analysis of multiple syncope clinical decision rules using a local data set†	NA	NA	NA
Trends in US physician work hours and career satisfaction	3.7 (0.6)	2.5 (2.1)	4 (1)
Does day of admission predict length of stay to Boise VAMC Inpatient Service?	4.3 (0.6)	3 (2)	4 (1)
Correlation between body mass index, chemotherapy dose and toxicity in breast cancer patients	5 (0)	4 (0)	4.3 (0.6)

* Based on the project review by three senior internal medicine faculties who are not participants in the curriculum. Rated on a 1–5 Likert scale.

† Reviews of this proposal are not available.

## Discussion

The curriculum is a work in progress, and it has demonstrated many lessons. First, we found that asking trainees to develop a project based on their experience and career interest, coupled with ongoing mentorship and protected time to pursue their work, are key elements to success. Substantial barriers remain, and include limited trainee time for regular meetings, faculty time for mentorship, and delays in IRB approval. Our assessment shows that residents demonstrate SBP and PBLI as part of this curriculum.

Other training programs have instituted curricula to demonstrate SBP and PBLI in the setting of residency training (3, 5, 6). A PubMed search revealed no longitudinal curriculum involving seminars, trainee-directed project development, faculty mentorship and work in progress meetings, dedicated trainee time for project development, and integration into the quality improvement for our medical center. In addition, we employ data managers and biostatisticians as project facilitators.

### Subsequent development

The 2011–2012 academic year brought about substantial change to our curriculum. As a result of a VA Center of Excellence grant in Primary Care Education, we integrated trainees from psychology, pharmacy, and nurse practitioner students into this course. We generated 23 project proposals, which exceeded our supply of faculty mentors. Through sequential voting, trainees and faculty ranked favored projects; we selected seven for further development. Trainees then opted into teams to continue work. We hoped interprofessional teams would coalesce to complete the year-long projects. This approach was only partially successful. Of the seven projects selected for development, only two have interdisciplinary team members. This was due to the medical subject matter of the projects and also in part due to the distribution of trainees in the curriculum: two psychology interns, two pharmacy interns, two nurse practitioner students, and 12 internal medicine residents. We maintain the structure of our seminar series and update the reading list annually based on the course director and faculty feedback.

Based on our ongoing experience with this interprofessional project, we aim to continue to make the interdisciplinary nature of teams an explicit requirement of project selection and team formation. Our goal is to instill team characteristics key to interprofessional collaboration including avoiding hierarchical structures, focus on problem solving rather than teamwork itself, shared goals, explicit complementary and interdependent roles, mutual respect, and power sharing (7, 8). This will be accomplished through background reading, written self-reflection, and faculty modeling. In addition, we will attempt to overcome scheduling hurdles, which are exacerbated by having different training schedules and increase collaboration via a web-based curriculum and a working document as part of an online ‘wiki’. This web-based tool will allow asynchronous communication between team members and mentors, serve as a forum for project discussion, and provide links to useful resources; such systems have been successfully used in large medicine residencies and nursing programs (9, 10). To our knowledge, research has not been published regarding their use in interdisciplinary health professional projects. We further focused project topics in areas that better lend themselves to interdisciplinary teams. These tended toward outpatient foci including medication reconciliation for complex patient, high-need/utilizing patients with multiple chronic illnesses, and reviews of interprofessional competencies. By using a menu of preselected topics and a simple rule that each team consists of three members from at least two different disciplines, we hope to further solidify interdisciplinary work. Finally, by offering routes in research and quality improvement paradigms, we are better able to offer projects that appeal to trainees of different backgrounds. In our experience, most pharmacy and nurse practitioner trainees have some exposure to quality improvement projects, while medicine residents and psychology trainees have done IRB-approved research.

As the Next Accreditation System will also include SBP and PBLI, we anticipate that the course will continue to meet ACGME requirements for these competencies and may allow us to better define our measures for successful implementation through ordered milestones (11).

### Limitations

Our survey included a self-assessment. We did not include a pretest for health systems knowledge. Our faculty rating tool has not been validated. The unique context of our medical center and resources available to trainees may not be available elsewhere.

## Conclusion

A structured curriculum provides an ongoing opportunity for demonstrating SBP and practice-based learning. Trainees gain knowledge and skills, and hospitals gain access to trainees who help to improve quality and meet accreditation requirements.
